# Hospital and Institutional News

**Published:** 1919-02-22

**Authors:** 


### HOSPITAL AND INSTITUTIONAL NEWS.

### PARLIAMENTARY MEDICAL COMMITTEE.

The recently formed House of Commons Medical Committee has appointed Sir Watson Cheyne chairman, and Major Farquharson secretary. Sir William Whitla, Lieut.-Col. N. Raw and Capt. Elliott constitute the Executive Committee. The Committee, as a whole, consists of members of the House possessing a medical or surgical degree, or who, in a particular way, are identified with medical or scientific matters. Exchange of views will take place among members of the Committee upon proposed legislation bearing on medical or allied questions, the object being to avoid expressing conflicting views in the course of Parliamentary debate. Further, the Committee will work in conjunction with medical and scientific bodies in the consideration of reports to be presented from time to time.

### SOME MORE EARLY ANNUAL REPORTS.

We have not, to date, received a single annual report for 1918 of a London hospital, although several from the provinces have been noticed in these columns and others are reaching us from day to day. When an institution of the importance of the Glasgow Royal Infirmary can issue complete accounts and statistics for the preceding year on February 3, an example is set which is difficult for the London secretary to emulate. We notice that this great Scottish institution was called upon last year to spend every penny of the £25,797 received from legacies. There has been much prosperity on the banks of the Clyde during the last four years, and it would have been only in accordance with national characteristics if some part of the revenue from legacies could have been treated as capital. Another early report is that of the Blackburn and East Lancashire Royal Infirmary, where the cost per occupied bed last year was £96 4s. 9d., a figure which, we venture to predict, will be below that of a great many hospitals. The report of the Aberdeen Royal Infirmary has also reached us, but the accounts are not appended. Proofs of the accounts of the Warneford Leamington Hospital and the North Ormesby Hospital, Middlesbrough, have also come to hand. We learn that at the last-named institution an appeal for increased weekly contributions from workmen has been a great success, and the secretary says that the hospital has received splendid support from the people of Middlesbrough (particularly the working class) during a most critical period.

### WOLVERHAMPTON'S RESPONSE.

Four years ago the Hospital Saturday Fund contribution to the General Hospital, Wolverhampton, amounted to £4,889. In 1915 an appeal was made to the working people of the town and district to increase their contributions to the fund. The appeal has had the happiest results. The balance sheet for 1918 shows the contributions to be £8,129 3s. 7d. After deducting £132 8s. for expenses, the balance has been allocated as follows:—General Hospital, £7,896 15s. 7d.; Penn Convalescent Home (attached to hospital), £100. This amount represents an increase of £3,240 on the sum contributed in 1914. At the annual meeting of the Fund a rousing appeal was made to members to endeavour this year to raise £10,000; if the increase on last year's figure should be 100 per cent. the hospital could do with the money. The Mayor (Councillor Jeffs) expresses the hope that the deficit of the hospital's current account, amounting to £4,000, will soon be extinguished. It is in contemplation to build a modern laundry and a new out-patient block, with up-to-date and special departments.

### THE TRIALS OF A LONDON DISPENSARY.

After most of the air raids on London official optimists usually endeavoured to soothe the public with the assurance that the damage done was "inconsiderable." This is a relative term, and certainly it does not apply to the case of the Royal General Dispensary in Bartholomew's Close. The building was damaged on two occasions, which rather upsets the theory, founded on the law of averages, that the safest place is where a bomb has already fallen. The first visitation was in September 1915, when the cost of repairing the damage done was met by insurance, and the second in 1917, when the destruction wrought was so extensive that a year elapsed before the premises were restored, much inconvenience being caused to both patients and officials. Incidentally, it may be remarked that the Rev. C. O. Becker is of opinion that changes in the methods of the dispensary, which would involve a policy of reconstruction, are needed to meet the social developments of the last fifty years. Before taking any steps the Committee are waiting to see whether reorganisation under Government direction may not be an event of the near future.

### WHITGIFT'S HOSPITAL THREATENED.

The friends of Whitgift's Hospital, Croydon, who in the past have successfully repulsed attacks which threatened to improve that ancient pile out of existence, are again called upon to be watchful. Two proposals are mooted. The first, which is described as "unofficial," is to demolish the south and west wings and throw a portion of the now enclosed quadrangle into the street. In the foreground, it is suggested, a war memorial should be erected, and a further detail of the plan is to place a cenotaph in the chapel, on which would be enshrined the names of the fallen. The wings left standing are in contemplation for use as a war museum. The scheme is described as having had a "chilly reception" when brought before the Borough Council, and, as it is not recognised by the Special Committee which has the war memorial in hand, probably it will come to nought. More danger threatens from the street widening at this spot, for which a committee of the Council has been authorised to prepare plans. Improvements which threatened the existence of the hospital have been rejected by the Local Government Board in the past, and any new demolition project would at once call to arms all defenders of one of our ancient monuments.

### REVISION OF MEDICAL TEACHING.

In a review of the situation in Birmingham an eminent medical authority points out that teachers in the medical schools up to the present have been receiving the veriest pittance, while they are not paid for their hospital work at all. What is most needed at present is a sufficient payment in the University for the chairs of medicine, surgery and midwifery, so that the occupants can be expected to give at least half their time to teaching. The next thing is the development of laboratories for pathology, bacteriology and kindred sciences, and these suitably organised for research. Further a chair should be devoted to preventive medicine, hygiene and public health, filling a gap much too common in medical teaching. Closer co-ordination of the work in the various Birmingham hospitals is particularly appealed for, and the whole list of weaknesses and discrepancies pointed out appears to be applicable not only to the midland town, but to most of the British medical teaching centres.

### EASING OF THE RATIONING SCHEME.

Hospital housekeepers and stewards will welcome the new regulation of the Food Controller that, as from February 9, institutions will not be called upon to collect coupons in respect of meals served, except in the case of persons residing in the establishment for six nights or more. This will remove the necessity of taking coupons from clerks, dispensers, and like workers, or of giving them invariably meatless meals. It will also simplify the keeping of the register of consumption in cases where the institutions have not obtained exemption in respect of this task. The general effect in most hospitals will be that the main meat supply will be made to feed a few more people, for we observe in the new regulations that the quantities of meat permitted by the various institutional scales at present in force at institutions will remain unaltered. Another useful concession is the removal of all restriction as to the purchase of lard and edible fats (other than nut butter). As the amount of dripping to be obtained from present supplies of meat is often disappointing, this facility for buying a substitute for use in pastry-making will be very helpful to housekeepers.

### HEALTH VISITORS IN CALCUTTA.

In discussing the annual report of the Municipal Administration of Calcutta at the Municipal Council, Sir Kailash Chandra Bose, referring to the Corporation midwives and lady health visitors, said:—"It is gratifying to note that the mortality of Calcutta is the lowest on record. The maternity work introduced by Dr. Crake has been able to establish its reputation in the northern quarters of the town amongst the poorer classes. It is hoped that when the work is well established, it will be possible to get thoroughly qualified persons; it would then be considered a boon to the busted population. It is imperative that our lady health visitors should go round the bustees, teach the people the elementary lessons of domestic hygiene, and try to impress upon the ignorant mind the necessity of neatness and cleanliness. The people need to have impressed upon them that erroneous feeding and neglect of hygienic measures make their convalescence prolonged and are very often the causes of premature death in their infants."

### TAXING DONATIONS TO HOSPITALS.

Should donations and subscriptions to hospitals and similar institutions be subject to income tax? This is not a new bone of contention, but in days of reconstruction the Committee of the Manchester and Salford Medical Charities (Hospital Sunday Fund) think the time is opportune to once more bring it to the front. They have accordingly forwarded a protest to the Prime Minister, the Chancellor of the Exchequer, Mr. Balfour, and Mr. Bonar Law, pointing out at the same time that the splendid medical service rendered by the voluntary institutions is all in the interest of the physical redemption of the people for the benefit of the nation at large. It does seem strange, not to say stupid, when *mens sana in corpore sano* has become one of the fashionable cults of the times, that a hard-hearted Chancellor of the Exchequer should levy tribute at the sources of its existence.

### TWILIGHT SLEEP: AN ECCLESIASTICAL COMMENT.

In connection with the justification of "twilight sleep" treatment, it may be of interest to quote the words of a very eminent Doctor of Divinity at one of our most famous universities. They come as a reply to a letter addressed by the author of the article published on another page, and it is hoped that some of the questions they suggest are to a certain small degree answered in that article. "I do not think that one can say that there is anything wrong in women avoiding the pains of child-birth by any means that is not wrong and hurtful. On the other hand, I do not think that they can claim as a right that means be used to prevent them from suffering these pains. So far I feel pretty clear in regard to two questions often asked. The main question in the letter, which raises the specific practical point, seems to me much more difficult than the general question. It is really a matter which needs very careful consideration by doctors and priests together. So far as my own opinion goes, I do not feel that one can rightly say that it is wrong for a woman to use means otherwise innocent which slightly increase the danger to herself while preventing pain. The risk to the child seems to me much more against the practice; I do not see how it can be right for the woman to purchase immunity from pain for herself at considerable risk to the child. In Capellmann's *Medicina Pastoralis* (pp. 38-40, edition 2), writing before the methods which are now becoming popular came up, he decided that the use of chloroform in ordinary cases of childbirth was not lawful because of the possible injury to the woman or to the child. I have heard it maintained that he was wrong in this on the ground that the slight administration of chloroform such as was at one time a good deal used in ordinary cases of childbirth was not likely to produce any considerable amount of injury to either. My impression is that the new methods have a greater risk so far as the child is concerned. But I think the whole matter needs very careful consideration by those who really understand the physical effects. Of course the administration of narcotics for obstetrical operations is a quite different question." Chloroform has now been so widely used in obstetrics with, in competent hands, no great admitted disadvantages that its employment may escape comment. But we think that, risk to the mother apart, in the case of "twilight sleep" considerable relative danger to the child is quite definitely proved, and should, therefore, very largely bring the views of both "doctors and priests" into line.

### GUM ARABIC IN SHOCK.

The introduction of this therapeutic agent by the eminent physiologist, Professor Bayliss, is of exceptional interest. As regards its efficiency in cases of shock, one may say that the *British Medical Journal* is convinced that "many patients were saved who, without injection, would certainly have died of their original injury or the necessary subsequent operation"—a more than favourable comment. But, although for many methods of treatment equal success has been claimed, in this instance it is the simplicity and the possibility of the widest application which appeal. Injection of normal saline has now been long practised (but with known limitations) and the introduction of a solution so simply prepared and requiring no complication of apparatus is assured a welcome. Medicine owes Professor Bayliss and the science he represents yet another debt of gratitude.

### THE INFLUENZA GERM.

The reported discovery of the causal germ in influenza has been hailed with such acclamation that it is well to remember that this, though a remarkable step, is nothing—as far as treatment goes—by itself. To combat the disease we must have two things. First, some agent which will act in a curative way on the healthy person as a prophylactic. Of the former, the antitoxic sera used in diphtheria give an example; of the latter, the anti-typhoid vaccine used in the Army to-day. The success of both is more than proved, but the germs were, in both cases, known for a considerable time before the remedial agents were developed. With influenza and the other common fevers recently mentioned with it, there is a definitely filterable virus and a supposed germ of extremely minute size. Probably its peculiarities do not cease here, and we must expect a great deal more work to be done on this subject before these diseases are placed in company with the select few practically mastered by mankind.

### THE PUBLIC AND "MARVELLOUS SURGERY."

One might rightly suggest that the extracts from the *Medical Supplement* compiled by the Medical Research Committee, recently published in the lay press, are more than a little misleading if the object be to bring before the public the excellence of British surgery in the war. Cases are instanced where toes are substituted for fingers and *vice-versa*, but surely these performances, depending upon little more than asepsis, patience, and common orthopædic technique, are not nearly so "marvellous" as the innumerable cases of critical operation which have withdrawn many a Britisher from the very jaws of death. Far greater development has occurred, and far finer work accomplished in vital surgery of the head, chest, and abdomen than in such purely reconstructional work.

### NOTTINGHAM'S PURE MILK CRUSADE.

Major the Hon. W. Astor, M.P., the new Parliamentary Secretary of the Local Government Board, suggested recently that "farmers or retailers who did not take the trouble to provide milk of a standard cleanliness should be paid less than the standard price." The hope was expressed in these columns that the authorities would take a braver course than this, and at least press for heavy fines in cases of "contaminated, dirty, tuberculous or stale milk." Nottingham for one is of our opinion. A warning has been issued to milk producers and dealers that "stern measures" are about to be taken to stop pollution. The condition in which milk is sent into the town indicates, in the opinion of the medical officer of health, "culpable carelessness." No amount of straining, he says, will remedy the evil. Proceedings are already being taken against offenders where pollution exists above a certain degree, and drastic action will follow if the scandal does not cease. One of the tricks of the trade to which the Medical Officer calls attention—a "growing practice" he describes it—"is to mix stale milk with the fresh." At the price now paid farmers and dealers for milk there should at the least be an approximation to purity. Milk should be health-giving not health-destroying.

### DENTAL TREATMENT FOR MOTHERS.

In connection with Worthing's maternity and child welfare scheme the Health Committee has decided that dentures be provided in necessitous cases where the medical officer is satisfied that such provision will materially conduce to the improvement of the health of the expectant mother. The quotation for supplying such dentures recommended for acceptance is: Full or nearly full (upper or lower), £2 to £3; partial (upper or lower), 10s. to £3; remodelling old dentures (upper or lower), 10s. to £1 10s.; repairing and adding to dentures, 5s. to £1. The maximum for one person is not to exceed £6, except in special cases requiring many fillings and two partial dentures.

### HEALTH AND TRADE UNIONS.

The National Association of Trade Union Approved Societies recently held its annual meeting in Manchester. The president, Mr. S. Sanderson, explained more than one grievance of the Association and most of these appeared to have their origin in that very prevalent desire—the demand for greater funds. The result of the war had been to throw a number of weakened men upon the care of the societies, and they claimed that the burden should be borne by the whole community and not by that section in receipt of wages less than £160 a year. Separate societies were uneconomical and it was suggested that all should be amalgamated into one association dealing with all concerned in the trade union movement, and that the Health Insurance Commissioners should give extra allowance to aid effective administration. It is a little hard to see why the trade unionist should demand aid from outside when his whole system is built up on self-protection. Undoubtedly, if all Health Insurance were completely under State control, as, indeed, these delegates suggest, there would be equality of treatment, but it is doubtful whether the health of the trade unionist or the cost of its civil supervision have suffered through the war in excess of other sections of the population.

### EARLIER MARRIAGE.

Interesting evidence was this week given by Dr. E. B. Turner to the National Birth-Rate Commission in London. The present economic situation had delayed the married phase of life to the extent of a number of years and these represented a time when the instinct of sex is probably at its strongest. In his opinion the crux of the whole question of venereal disease lay in earlier marriages and a resultant raising of the moral standard throughout the country. Recently the greater freedom and dispersal of women, many of very tender years, had, in conjunction with the wave of sentimental patriotism passing over the country, developed a laxity which it would take a long time to overcome without considerable education and care. Dr. Turner gives many instances in support of his view, and it must be recognised that with these scourges we are not dealing with a problem for the physician alone, but with one where the true cause lies in the deepest ramifications of national economy. Delay in marriage is not a product of the war alone. It has been steadily increasing for many years.

### PUBLIC PHARMACISTS.

Mr. A. H. Jenkin presided over a representative gathering of pharmacists engaged in voluntary hospitals, dispensaries, and kindred institutions at the annual meeting of the Public Pharmacists' Association. The report stated that the advance made by negotiations with the Army authorities, whereby the civil qualification of a pharmacist is now recognised as being necessary to any person placed in charge of a hospital of 100 beds or over, was regarded with satisfaction. Dispensing by unqualified persons at camp hospitals had also received the attention of the Council. Twelve new members were elected, and it is noteworthy to record that eleven of these were qualified women pharmacists. Practically every public dispensary service is represented on the new Council, the voluntary hospital representation being stronger than previously. The chairman appealed for a larger measure of support from the general hospitals. There was, he said, a demand for the *practical* teaching of pharmacy so that pharmacists taking up hospital appointments might be well equipped for manufacturing the various preparations required in the laboratories of those institutions. This specialised teaching would grow in insistence when the new schemes on health were launched.

### WAR BONUS AND FRAUDS.

The first case to reach the public of a successful fraud in connection with the payment of war bonus has come before the Bristol Police Court. It concerned a non-resident attendant, who had since left the employment of the Bristol Poor Law Guardians. The offence consisted in the misrepresentation of the ages of the man's children, whereby he received £8 4s. 6d. more than the sum to which he was entitled. Since his friends offered to repay the loss to the Guardians, and the man, an ex-constable, had suffered from sun-stroke, the Bench bound him over for twelve months in the sum of £5, and a surety of £5. In spite of the multiplicity of forms such cases have been rare, but perhaps the circumstances of non-resident employees renders them more liable to temptation than is the case with those whose homes are in the institutions.

### THIS WEEK'S DRUG MARKET.

The downward tendency of prices which began almost immediately after the signing of the armistice, is still the main characteristic of the drug market. Dealers in fact now appear to have become resigned to this condition, and one seldom hears the opinion expressed that prices will advance again. Two months or so ago, this view was freely voiced; possibly the wish was father to the thought. Here and there are drugs which have a slightly dearer tendency, and there are, of course, many cases in which prices are maintained, but the bulk of the fluctuations are in a downward direction. The cheapest offers of sugar of milk have been cleared and temporarily more money has to be paid, but it is difficult to see how this condition can be maintained without recourse to artificial expedients. Bromides are offered at substantially lower rates. Other drugs which are cheaper are carbonate of ammonia, barbitone, aspirin, benzoic acid, sodium benzoate, carbolic acid, salicylic acid, sodium salicylate, chloral hydrate, cream of tartar, calumba root, gamboge, honey, artificial oil of wintergreen, phenacetin, saccharin and senegar root. Among those which have an easier price tendency may be mentioned Persian opium, myrrh, salol, aniseed oil, Indian hemp, clove oil and balsam copenta.

